# Investigating the role of platelets and platelet-derived transforming growth factor-β in idiopathic pulmonary fibrosis

**DOI:** 10.1152/ajplung.00227.2022

**Published:** 2023-08-29

**Authors:** Deborah L. W. Chong, Theresia A. Mikolasch, Jagdeep Sahota, Carine Rebeyrol, Helen S. Garthwaite, Helen L. Booth, Melissa Heightman, Emma K. Denneny, Ricardo J. José, Akif A. Khawaja, Anna Duckworth, Myriam Labelle, Chris J. Scotton, Joanna C. Porter

**Affiliations:** ^1^UCL Respiratory, Division of Medicine, University College London, London, United Kingdom; ^2^Institute for Infection and Immunity, St George’s University of London, London, United Kingdom; ^3^Interstitial Lung Disease Service, University College London Hospital, London, United Kingdom; ^4^Department of Clinical and Biomedical Science, University of Exeter, Exeter, United Kingdom; ^5^Department of Developmental Neurobiology, St. Jude Children’s Research Hospital, Memphis, Tennessee, United States

**Keywords:** inflammation, interstitial lung diseases, platelets, pulmonary fibrosis, transforming growth factor-β1

## Abstract

Transforming growth factor-β1 (TGFβ1) is the key profibrotic cytokine in idiopathic pulmonary fibrosis (IPF), but the primary source of this cytokine in this disease is unknown. Platelets have abundant stores of TGFβ1, although the role of these cells in IPF is ill-defined. In this study, we investigated whether platelets, and specifically platelet-derived TGFβ1, mediate IPF disease progression. Patients with IPF and non-IPF patients were recruited to determine platelet reactivity, and separate cohorts of patients with IPF were followed for mortality. To study whether platelet-derived TGFβ1 modulates pulmonary fibrosis (PF), mice with a targeted deletion of TGFβ1 in megakaryocytes and platelets (TGFβ1^fl/fl^.PF4-Cre) were used in the well-characterized bleomycin-induced pulmonary fibrosis (PF) animal model. In a discovery cohort, we found significantly higher mortality in patients with IPF who had elevated platelet counts within the normal range. However, our validation cohort did not confirm this observation, despite significantly increased platelets, neutrophils, active TGFβ1, and CCL5, a chemokine produced by inflammatory cells, in the blood, lung, and bronchoalveolar lavage (BAL) of patients with IPF. In vivo, we showed that despite platelets being readily detected within the lungs of bleomycin-treated mice, neither the degree of pulmonary inflammation nor fibrosis was significantly different between TGFβ1^fl/fl^.PF4-Cre and control mice. Our results demonstrate for the first time that platelet-derived TGFβ1 does not significantly mediate inflammation or fibrosis in a PF animal model. Furthermore, our human studies revealed blood platelet counts do not consistently predict mortality in IPF but other platelet-derived mediators, such as C-C chemokine ligand 5 (CCL5), may promote neutrophil recruitment and human IPF.

**NEW & NOTEWORTHY** Platelets are a rich source of profibrotic TGFβ; however, the role of platelets in idiopathic pulmonary fibrosis (IPF) is unclear. We identified that patients with IPF have significantly more platelets, neutrophils, and active TGFβ in their airways than control patients. Using an animal model of IPF, we demonstrated that platelet-derived TGFβ does not significantly drive lung fibrosis or inflammation. Our findings offer a better understanding of platelets in both human and animal studies of IPF.

## Graphical abstract

**Figure:**
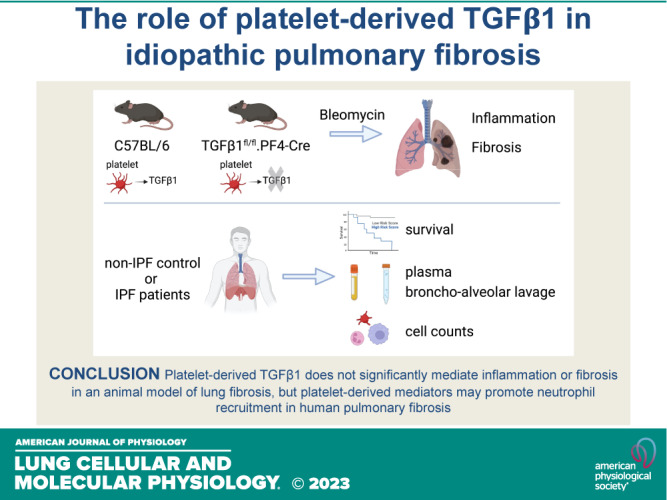

## INTRODUCTION

Interstitial lung diseases (ILDs) consist of over 200 inflammatory and fibrotic diseases affecting the lung interstitium (1), with idiopathic pulmonary fibrosis (IPF) representing the most severe of the idiopathic interstitial pneumonias (IIPs) (2). IPF is characterized by aberrant wound healing resulting from multiple microscopic sites of alveolar epithelial injury (3, 4). The histological hallmark feature of IPF lung is the presence of fibrotic foci, consisting of proliferative fibroblasts, differentiated myofibroblasts, and increased deposition of extracellular matrix (ECM) proteins such as collagen (4). These features contribute to loss of lung architecture and function and ultimately poor prognosis for patients with a mean survival of 3–5 yr from diagnosis (1). Two antifibrotic therapeutics, Pirfenidone and Nintedanib, have been approved for use in patients with IPF since 2014. However, these treatments merely slow disease progression (5), and there is currently no cure for this fatal disease. A better understanding of disease mechanisms is urgently required in the search for more effective treatments.

Transforming growth factor-β1 (TGFβ1) has a plethora of biological roles ranging from inflammation through fibrosis (6) and wound healing (7). In order for TGFβ to exert its functional effects, it must be activated from its latent form by dissociation from the latency-associated peptide (LAP) and latent TGFβ binding protein (LTBP). Activation of TGFβ is important in determining the availability of bioactive TGFβ to mediate its downstream biological effects such as fibrosis (6).

TGFβ1 is elevated in bronchoalveolar lavage fluid (BALF) of patients with IPF (8), and mouse pulmonary fibrosis (PF) models (9) and genetic polymorphisms in *tgfb* are associated with IPF disease progression (10). Fibroblasts stimulated with TGFβ1 have enhanced α-smooth muscle actin (αSMA) expression and collagen deposition, supporting the importance of this cytokine in differentiating fibroblasts into a fibrotic phenotype (11). However, the exact cellular source of TGFβ1 in IPF is unknown, although alveolar macrophages, bronchial epithelial cells, and PD-1^+^ TH17 cells have been proposed (12–14). Identifying the exact cellular source of TGFβ1 may allow cell-specific therapies in IPF. Such targeted therapy would have the advantage of avoiding global deletion of this multifunctional cytokine that is also required for other critical processes including immune regulation and tumor suppression.

Platelets are key mediators in immunity and tissue remodeling and represent an abundant source of TGFβ1, which can be released rapidly on cell activation (15). The role of platelets and their released mediators have been explored in several fibrotic and tissue-remodeling diseases. For example, during pulmonary tuberculosis infection, platelets promote inflammation and ECM degradation (16). Platelets also attenuate liver fibrosis by degrading ECM (17), although platelet-derived TGFβ1 has been shown to be pathogenic in cardiac or liver fibrosis (18, 19). This suggests that the biological roles of platelets and platelet-derived mediators such as TGFβ1 may be tissue specific.

The role of platelets in chronic respiratory conditions such as IPF is now actively being pursued (20). It has been reported that patients with IPF have elevated mean blood platelet volume (MPV) compared with healthy controls, and MPV may represent a surrogate marker for platelet activation in IPF (21). Patients with IPF also have augmented platelet reactivity in terms of increased CD62P (P-Selectin) expression and circulating platelet-monocyte aggregates (22). Murine models of bleomycin-induced PF have shown increased platelet trapping in the lungs, correlating with elevated collagen deposition (23). Furthermore, depletion of platelets in bleomycin-treated mice reduced fibrotic disease severity (24), highlighting a pathogenic role for platelets in pulmonary fibrosis.

Platelets and neutrophils act in a synergistic manner to cross the endothelium during inflammation in vitro and in vivo (25). Along with the potential profibrotic effects of platelets, platelets may also stimulate neutrophil recruitment into the lung as a result of lung injury or inflammation. Increased frequency of neutrophils within the BALF of patients with IPF has been shown to be an independent indicator of poor prognosis and released mediators such as neutrophil elastase and neutrophil extracellular traps (NETs) can mediate PF pathology (26–28). It is unclear whether platelets and platelet-derived mediators such as platelet-derived TGFβ1 can impact neutrophil recruitment from the blood into IPF lung to further potentiate the fibrotic process.

Despite the growing body of evidence demonstrating the increased presence of activated platelets in clinical and animal studies of IPF, it is still unclear how platelets drive fibrotic disease progression and whether platelets represent a major source of profibrotic TGFβ1 in IPF. Therefore, in this study we sought to define whether platelets, and more specifically, platelet-derived TGFβ1, mediate IPF disease progression using both clinical patient samples and an animal model of pulmonary fibrosis.

## MATERIALS AND METHODS

### Patient Samples and Mice

Human neutrophils, platelet-rich plasma (PRP), BALF or lung biopsies from male and female healthy controls, patients with IPF or non-ILD, were collected with written informed consent and research ethics committee approval (IRAS study I.D. 29531). For survival analysis, male and female patients with IPF from the University College London Hospital (UCLH) or Exeter ILD Services were followed prospectively for mortality based on blood platelet counts as described in Supplemental Methods (ethics study number REC reference 18/L0/0937). Blood platelet counts from a cohort of male and female patients with IPF or healthy controls were obtained from the UK Biobank (applications 9072 and 44046). Patient demographics are shown in Table 1 and in Supplemental Data. TGFβ1^fl/fl^.PF4-Cre mice were obtained from Prof. Richard Hynes (29) and maintenance and procedures were approved and conducted in accordance with UK Home Office Regulations (PPL no. 70/8125).

**Table 1.** Summary of cohort of patients with IPF for prospective mortality study

Cohort	Platelet Strata	Mean Age, yr ± SD	Mean FVC, % Predicted ± SD	% Male
UCLH	Group 1 (*n* = 71)	73.3 ± 10.3	77.9 ± 21.8	77.5%
	Group 2 (*n* = 72)	73.9 ± 8.4	75.9 ± 18.6	86.1%
	Group 3 (*n* = 71)	72.8 ± 8.3	73.4 ± 19.8	74.7%
Exeter	Group 1 (*n* = 94)	74.1 ± 8.9	83.0 ± 17.0	89.0%
	Group 2 (*n* = 96)	71.6 ± 8.9	83.0 ± 20.0	81.0%
	Group 3 (*n* = 95)	74.0 ± 8.5	84.0 ± 19.0	57.0%

FVC, forced vital capacity; IPF, idiopathic pulmonary fibrosis; UCLH, University College London Hospital.

### Patient BALF and Plasma Sampling

Patient BALF and plasma were collected and processed as described in Supplemental Methods. Cell counts were performed on cytospun BALF (*n* = 6–12). BAL cells were stained with anti-CD61 antibody (clone VI-PL2, BD Biosciences, Cat. No. 555754, RRID:AB_396095) or mouse IgG1 isotype control (clone MOPC-21, BD Biosciences, Cat. No. 555754, RRID:AB_396095), and percentage of CD61^+^ platelets in BALF (*n* = 3–6) was quantified on a BD FACSVerse flow cytometer (BD Biosciences, San Jose) and analyzed with FlowJo v10 software (FlowJo LLC, Ashland, RRID:SCR_008520).

### Cytokine Analysis

Total TGFβ1, CCL5, C-X-C chemokine receptor type 4 (CXCR4), and total matrix metalloproteinase-7 (MMP7) were quantified in PRP (*n* = 4–5), plasma (*n* = 10–37), and BALF (*n* = 7–16) using ELISA kits (R&D systems, Minneapolis). Active TGFβ1 was quantified by a Mink lung epithelial cell (MLEC) bioassay (30) as described in Supplemental Methods.

### Neutrophil Chemotaxis Assay

Human or murine neutrophils and PRP were isolated as described in Supplemental Methods. In brief, untreated, 1 μM SB-525334 (Sigma-Aldrich, Gillingham, UK), 10 μM Galunisertib (Ly2157299, Antibodies-online GmbH, Germany) ALK5 inhibitors or DMSO vehicle pretreated neutrophils (*n* = 4) were added to the top of 8 μm PET trans-wells (Corning, New York) and chemoattractants: 100 nM *N*-formylmethionine-leucyl-phenylalanine (fMLP) (Sigma-Aldrich), 1 ng/mL TGFβ1 (R&D systems), 10 ng/mL leukotriene B4 (LTB_4_) (Cambridge Biosciences, Bar Hill, UK) or PRP added to the bottom. After 1 h at 37°C, cells that had migrated toward the chemoattractant in the bottom chamber were counted on a BD FACSVerse flow cytometer.

### Bleomycin Animal Model of Pulmonary Fibrosis

Male and female TGFβ1^fl/fl^.PF4-Cre or littermate control mice (11–24 wk old; *n* = 4–15) were randomized into treatment or control groups. The treatment groups were given 25 or 50 IU bleomycin (Kyowa Hakko Kinn UK Ltd, Slough, UK) in a volume of 50 μL 0.9% saline by oropharyngeal instillation. The control group was given 50 μL 0.9% saline by oropharyngeal instillation. Mice were monitored for disease severity and culled at 6, 21, and 28 days post instillation. Individual mice were coded for each experiment to allow for blinded result assessments and data analysis.

### Quantification of Murine Pulmonary Inflammation

BALF was obtained from murine lungs by three washes with 1 mL PBS. BALF was centrifuged and cell pellet was processed for cytospin cell counts. Murine lungs were homogenized in PBS before red blood cell lysis. Cells were blocked with Fc block (BD Biosciences, Cat. No. 553141, RRID:AB_394656) and stained with anti-Ly6G-PE (clone 1A8, BD Biosciences, Cat. No. 551461, RRID:AB_394208), anti-CD11b-APC (clone M1/70, BD Biosciences, Cat. No. 553312, RRID:AB_398535), and anti-CD11c-APC-H7 (clone HL3, BD Biosciences, Cat. No. 561241, RRID:AB_10611727) antibodies. Cells were analyzed on a BD FACSVerse flow cytometer and FlowJo software.

### Quantification of Murine Pulmonary Fibrosis

Dehydrated murine lungs were scanned on a SkyScan-1072 micro-CT scanner and images were reconstructed using SkyScan NRecon software (both from Bruker MicroCT, Coventry, UK). The degree of fibrosis in the lungs was assessed using InForm image analysis software (Perkin Elmer, Massachusetts) as previously described (31). Total lung collagen was quantified via the measurement of hydroxyproline by reverse-phase high-performance liquid chromatography (HPLC) (32).

### Histology and Immunohistochemistry

Murine or human lungs were processed for histological analysis as described in Supplemental Methods. Immunohistochemistry (IHC) was performed for murine (clone AB-773, 20 µg/mL, Sigma-Aldrich, Cat. No. SAB4300361, RRID:AB_10621932) or human CD61 (clone 2f2, 550 µg/mL, Leica Biosystems, Wetzlar, Germany). Sections were scanned on a Nanozoomer Digital Slide Scanner and analyzed using NDP.view software (both from Hamamatsu Corporation, Hamamatsu City, Japan). Quantification of collagen deposition in alveolar walls based on modified Martius Scarlet Blue (MSB) staining was performed using Orbit Image analysis software (33).

### Statistical Analysis

All experiments were conducted with at least three technical replicates. For neutrophil experiments, three to four biological replicates were used to minimize donor variability. All statistical analyses with the Gehan–Breslow–Wilcoxon test, linear regression, Mann–Whitney *U* test, unpaired *t* test, one- or two-way ANOVA with Holm–Sidak post hoc testing were performed with Prism v9 software (GraphPad, San Diego, RRID:SCR_002798). Cox proportional hazard modeling, with multivariate analysis using mortality data of patients with IPF, was conducted using OriginLab statistical software. Unless stated otherwise, means ± SE are shown in all graphs with a *P* < 0.05 considered significant.

## RESULTS

### Blood Platelet Counts Do Not Consistently Predict Mortality in IPF

**Figure 1.**
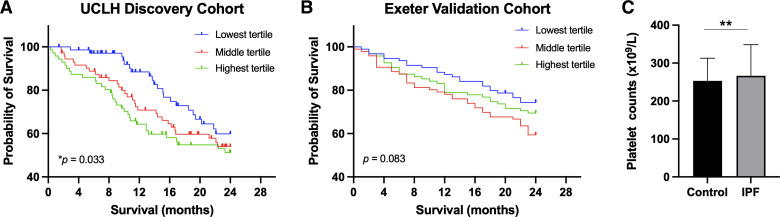
Human blood platelet counts do not consistently predict mortality in IPF. *A* and *B*: Kaplan–Meier survival curves of patients with IPF from two different cohorts: UCLH discovery cohort (*n* = 214 patients; *A*) or Exeter validation cohort of patients with IPF (*n* = 285 patients; *B*). Patients were divided into three strata based on blood platelet count (see Supplemental Methods for blood cutoff values for tertiles). The three strata were defined bias-free by dividing into three equal groups: lowest tertile (*blue*), middle tertile (*red*), and highest tertile (*green*). *C*: blood platelet counts of patients with IPF (*n* = 162) or healthy controls (*n* = 3,77,093) from UK Biobank data. Means ± SD are shown. Any statistical differences between survival curves or blood platelet counts were determined using the Gehan–Breslow–Wilcoxon test or unpaired *t* test (**P < 0.01). IPF, idiopathic pulmonary fibrosis.

We initially sought to investigate the role of platelets in IPF disease progression. A discovery cohort of consecutive, unselected patients with IPF (Table 1) from UCLH ILD Services was prospectively followed for mortality. Patients were split into three equally sized cohorts based on platelet count using an unbiased approach (see Supplemental Methods for cutoff values). Mortality was highest in patients with the highest platelet count (>267 × 10^9^/L) and lowest in those with the lowest platelet counts (<207 × 10^9^/L, *P* = 0.033; Fig. 1*A*). However, this observation using similar methodology was not reproduced in a larger validation cohort of patients with IPF from Exeter ILD Services (Fig. 1*B* and Table 1), where there was no significant correlation between blood platelet counts with risk of mortality (*P* = 0.083). In addition, data from the UK Biobank revealed that patients with IPF had significantly higher blood platelet blood counts than healthy controls (Fig. 1*C*, *P* < 0.01).

Cox proportional hazard modeling with multivariate analysis was conducted to further investigate any potential correlations with mortality in the UCLH and Exeter patient cohorts. In both the UCLH and Exeter cohorts, higher percentage in forced vital capacity (FVC) was found to be significantly protective (probability for hazard ratio was 0.005 for UCLH cohort and 0.0001 for Exeter cohort). In addition, age at diagnosis was found to offer protection in the Exeter cohort, but not in the UCLH patient cohort (probability for hazard ratio was 0.0272 and 0.7808, respectively). Cox proportional hazard modeling based on sex or platelet counts did not show any significant correlation with mortality in either patient cohort. These findings suggest that although blood platelet counts do not consistently predict IPF disease progression or survival, patients with IPF have significantly more platelets in their blood than healthy controls.

### Active TGFβ1 Levels Correlate with an Activated Blood Platelet Signature in Patients with IPF

**Figure 2.**
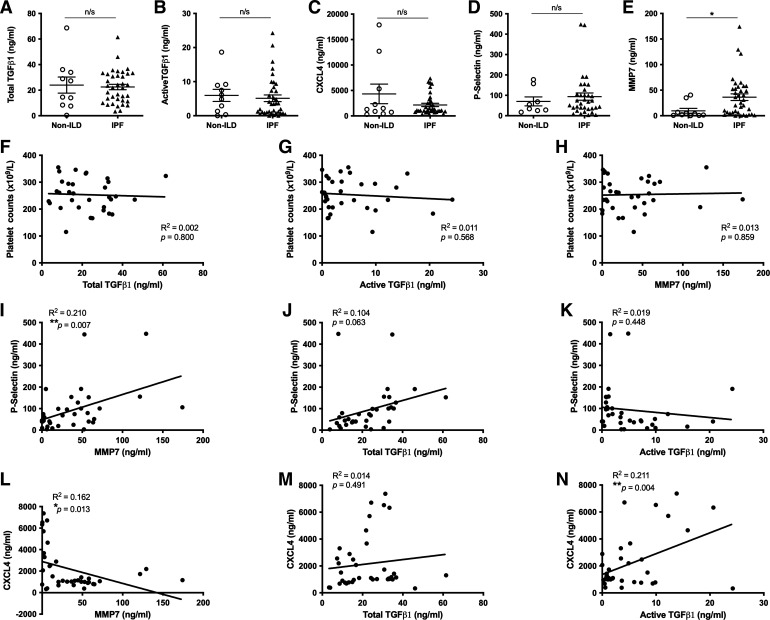
Levels of active TGFβ1 correlate with an activated blood platelet signature in patients with IPF. Concentrations of total (*A*) or active TGFβ1 (*B*), CXCL4 (*C*), P-Selectin (*D*), and MMP7 (*E*) were measured in the plasma of patients with non-ILD and IPF (*n* = 10 and 37, respectively) by ELISA or MLEC bioassay. Concentrations of total (*F*) or active TGFβ1 (*G*) or MMP7 (*H*) in the plasma of patients with IPF were correlated with paired blood platelet counts (*n* = 33). Concentrations of MMP7 (*I*), total (*J*), or active TGFβ1 (*K*) in the plasma of patients with IPF were correlated with paired P-Selectin levels (*n* = 37). Concentrations of MMP7 (*L*), total (*M*), or active (*N*) TGFβ1 in the plasma of patients with IPF were correlated with paired CXCL4 levels (*n* = 37). Any statistical differences were determined using unpaired *t* test or linear regression and any significant differences are indicated (n/s = not significant, *P < 0.05, **P < 0.01. IPF, idiopathic pulmonary fibrosis; MLEC, Mink lung epithelial cell bioassay; non-ILD, non-interstitial lung diseases.

Given that patients with IPF have significantly more circulating platelets than healthy controls (Fig. 1*C*), we next investigated whether mediators released by activated platelets were increased in patients with IPF and non-ILD (Supplemental Table S1). We found no significant difference in the levels of total or active TGFβ1 in the plasma of patients with non-ILD or IPF (Fig. 2, *A* and *B*). There was also no difference between concentrations of CXCL4 and soluble P-Selectin, two surrogate platelet activation markers (34, 35) in IPF plasma compared with non-ILD controls (Fig. 2, *C* and *D*). MMP7, a recognized IPF disease severity biomarker (36), was significantly elevated in the plasma of our cohort of patients with IPF compared with non-ILD controls (Fig. 2*E*, *P* = 0.044). In addition, we found no correlation in the levels of total or active TGFβ1 with blood platelet counts from patients with IPF (Fig. 2, *F* and *G*). MMP7 concentration did not correlate with blood platelet counts from patients with IPF (Fig. 2*H*), to further support our prospective mortality data showing blood platelet counts alone do not predict IPF disease progression (Fig. 1). However, concentrations of P-Selectin significantly correlated with increasing concentrations of MMP7 in the plasma of patients with IPF (Fig. 2*I*) to suggest that activated platelets may contribute to IPF disease, although levels of platelet-derived mediators such as TGFβ1 did not correlate with P-Selectin levels (Fig. 2, *J* and *K*). In contrast to our P-Selectin data, CXCL4 levels inversely correlated with MMP7 (Fig. 2*L*), although active TGFβ1 in IPF plasma positively correlated with CXCL4 levels (Fig. 2*N*). CXCL4 levels did not significantly correlate with paired total TGFβ1 levels (Fig. 2*M*). Overall, significant correlations between CXCL4 and active TGFβ1 identify activated platelets as a possible cellular source of this profibrotic cytokine in IPF.

### Platelet-Derived TGFβ1 Does Not Significantly Mediate Fibrosis or Disease Resolution in Bleomycin-Induced PF

**Figure 3.**
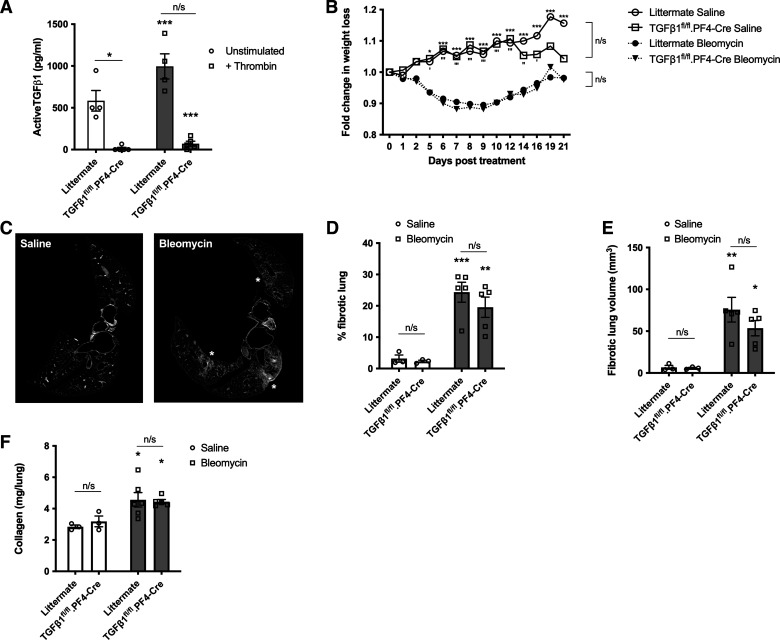
Platelet-derived TGFβ1 does not play a significant role in bleomycin-induced fibrosis. *A*: quantification of secreted active TGFβ1 by MLEC bioassay in unstimulated (*white* bars) or thrombin-treated (*gray* bars) PRP from littermate (*n* = 4) or TGFβ1^fl/fl^.PF4-Cre (*n* = 5) mice. *B*: TGFβ1^fl/fl^.PF4-Cre or littermate mice were given 50 IU bleomycin (*n* = 5/group) or saline (*n* = 3/group) via an oropharyngeal route. Fold-change in body weight loss was monitored for 21 days post instillation. *C*: representative micro-CT scans of lungs after 21 days of treatment (white asterisks denote fibrotic lesions). Percentage (*D*) or volume (*E*) of fibrotic lung tissue in micro-CT scanned lungs was determined using InForm analysis software (*n* = 3 saline/mouse group and *n* = 5 bleomycin/mouse group). *F*: total lung collagen of murine lungs was determined by reverse-phase HPLC (*n* = 3 saline/mouse group and *n* = 5 bleomycin/mouse group). Any statistical differences were determined using two-way ANOVA with Holm–Sidak post hoc testing or Mann–Whitney *U* tests. In B, asterisks represent significant differences between treatments in littermate control mice and apostrophes represent significant differences between treatments in TGFβ1^fl/fl^.PF4-Cre mice. In *D* and *E*, asterisks above the bars represent significance between saline and bleomycin treatment. Any other significant differences are indicated (n/s = not significant, *P < 0.05, **P < 0.01, ***P < 0.001). MLEC, Mink lung epithelial cell bioassay; PRP, platelet-rich plasma.

Platelets are known to be a source of TGFβ1 (15), which can be released on activation with agonists such as thrombin. Active TGFβ1 was readily detected in thrombin-activated human PRP (Supplemental Fig. S1). To investigate whether platelet-derived TGFβ1 has a direct profibrotic role in IPF, a conditional knockout transgenic mouse, in which TGFβ1 is deleted in megakaryocytes and subsequent platelets (TGFβ1^fl/fl^.PF4-Cre) (29) was used in the bleomycin-induced PF model. At baseline, unstimulated or thrombin-activated TGFβ1^fl/fl^.PF4-Cre PRP contained little or no active TGFβ1 compared with PRP from littermate controls (Fig. 3*A*), confirming the knockout phenotype.

Following 21 days after bleomycin (50 IU) treatment, littermate and TGFβ1^fl/fl^.PF4-Cre mice had regained initial weight loss with no significant difference between bleomycin-treated littermate or TGFβ1^fl/fl^.PF4-Cre mice (Fig. 3*B*). No difference was found between saline-treated groups (Fig. 3*B*). To determine the extent of fibrotic lung tissue, micro-CT image analysis based on tissue segmentation (31) revealed fibrotic lesions within the bleomycin-treated lungs (Fig. 3*C*). A trend of decreased percentage of fibrotic lung tissue and lung volume between bleomycin-treated littermate and TGFβ1^fl/fl^.PF4-Cre mice was observed (Fig. 3, *D* and *E*) but did not reach statistical significance (*P* = 0.3979 and *P* = 0.2669, respectively). HPLC analysis for hydroxyproline (a major component of collagen) content in whole lungs (Fig. 3*F*) confirmed that the loss of platelet-derived TGFβ1 did not impact the degree of fibrosis in this model.

**Figure 4.**
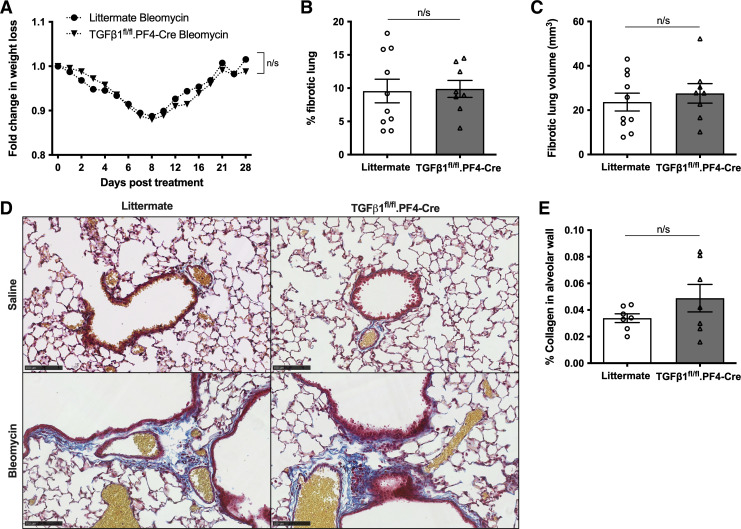
Platelet-derived TGFβ1 does not play a significant role in disease resolution after bleomycin-induced injury. *A*: TGFβ1^fl/fl^.PF4-Cre (*n* = 8) or littermate mice (*n* = 10) were given 25 IU bleomycin via an oropharyngeal route. Fold-change in body weight loss was monitored for 28 days post instillation. Percentage (*B*) and volume (*C*) of fibrotic lung tissue in micro-CT scanned lungs were determined using InForm analysis software (*n* = 10 littermate controls or *n* = 8 TGFβ1^fl/fl^.PF4-Cre mice). *D*: representative images of modified Martius Scarlet Blue (MSB) trichrome stained saline- (*top*) or bleomycin-treated (*bottom*) littermate control (*left*) or TGFβ1^fl/fl^.PF4-Cre (*right*) lung sections from two independent experiments (blue = collagen, yellow = RBC, red = cytoplasm, dark red = nuclei). Images are shown as ×20 magnification as denoted by 100 µm scale bar. *E*: quantification of percentage of alveolar collagen deposition in bleomycin-treated littermate control or TGFβ1^fl/fl^.PF4-Cre lungs based on MSB stained lungs. No statistical differences were found using two-way ANOVA with Holm–Sidak post hoc testing or Mann–Whitney *U* tests (n/s = not significant).

Aside from its profibrotic effects, TGFβ1 is also recognized to play a role in wound healing. A single administration of bleomycin at a lower dose allows the lung to heal after the initial injury. We next examined whether platelet-derived TGFβ1 modulates disease resolution after bleomycin-induced PF. At 28 days after a single bleomycin challenge with a lower dose (25 IU), the percentage weight loss between littermate and TGFβ1^fl/fl^.PF4-Cre animals did not differ (Fig. 4*A*). Micro-CT analysis revealed equivalent remaining lung fibrosis (Fig. 4, *B* and *C*). This suggests that platelet-derived TGFβ1 does not alter the rate of disease resolution. Histological staining of murine lung tissue revealed dense cellular infiltration (as shown by H&E staining) and abundant CD61 expression (a platelet and platelet aggregates marker) in inflamed and fibrotic lesions (Supplemental Fig. S2). Histological analysis revealed more ECM deposition in bleomycin-treated mice compared with saline controls as shown by blue modified MSB trichrome staining (Fig. 4*D*). No difference in percentage of collagen deposition in alveolar walls in either bleomycin-treated littermate or TGFβ1^fl/fl^.PF4-Cre animals was found when MSB trichrome stained images were digitally analyzed (Fig. 4*E*). This suggests the lack of platelet-derived TGFβ1 in TGFβ1^fl/fl^.PF4-Cre mice does not significantly affect ECM deposition following bleomycin treatment. These results establish for the first time that platelet-derived TGFβ1 does not significantly drive lung fibrosis or disease resolution in an animal mode of PF despite activated platelets providing an abundant source of profibrotic TGFβ1 ex vivo.

### TGFβ1 and Platelet-Derived TGFβ1 Are Potent Neutrophil Chemoattractants In Vitro

**Figure 5.**
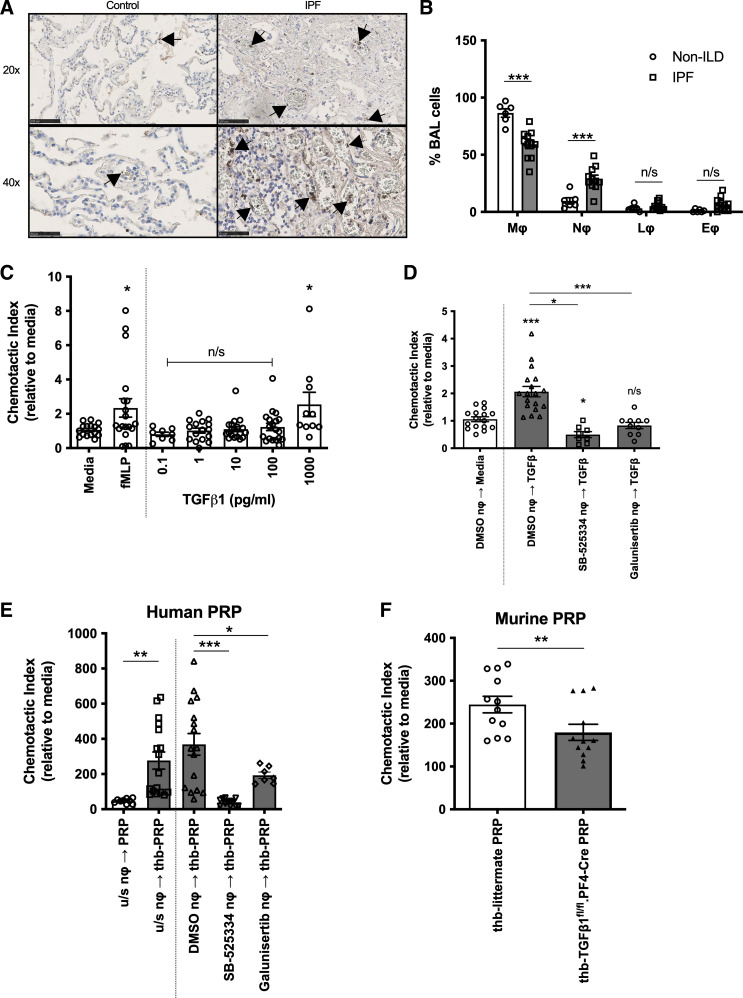
Platelet-derived TGFβ1 partially contributes to neutrophil chemotaxis. *A*: CD61^+^ platelets and platelet aggregates (indicated by the arrows) in control and IPF lung were detected by IHC. Size denoted by scale bar (×20 scale bar = 100 µm, ×4 scale bar = 50 µm). *B*: cell counts of macrophages (mϕ), neutrophils (nϕ), lymphocytes (lϕ), and eosinophils (eϕ) were determined in patients with non-ILD (*n* = 6) and IPF (*n* = 11) by cytospins of recovered cells in BALF. *C*: chemotaxis of human neutrophils (*n* = 4 healthy donors) toward media, 100 nM fMLP or increasing TGFβ1 concentrations (0.1–1,000 pg/mL). *D*: chemotaxis of DMSO, SB-525334 or Galunisertib ALK5 inhibitors pretreated human peripheral neutrophils (*n* = 3 or 4 healthy donors) toward media (white bar) or 1 ng/mL TGFβ1 (dark gray bars). *E*: chemotaxis of untreated, DMSO, SB-525334 or Galunisertib ALK5 inhibitors pretreated human peripheral neutrophils (*n* = 3 or 4 healthy donors) toward unstimulated (white bar) or thrombin (thb)-activated human PRP (gray bars). *F*: chemotaxis of murine bone marrow-derived neutrophils (*n* = 4 littermate mice) toward thrombin (thb)-activated littermate (white bar) or TGFβ1^fl/fl^.PF4-Cre (black bar) murine PRP. Statistical differences were determined using Mann–Whitney *U* test or one-way ANOVA with Holm–Sidak post hoc testing (n/s = not significant, *P < 0.05, **P < 0.01, ***P < 0.001). IHC, immunohistochemistry; IPF, idiopathic pulmonary fibrosis; non-ILD, non-interstitial lung diseases; PRP, platelet-rich plasma.

Increased CD61^+^ platelets and platelet aggregates were found within IPF lung, particularly in the blood vessels (Fig. 5*A*). Interestingly, we observed BALF from patients with IPF contained significantly more neutrophils than non-ILD controls (Fig. 5*B*). Platelets can aid neutrophil migration (25) but it is unknown whether platelets, via the secretion of mediators such as TGFβ1, can drive neutrophilic inflammation observed in IPF. We first assessed whether recombinant TGFβ1 can influence neutrophil migration in vitro. Human neutrophils migrated in a concentration-dependent manner toward TGFβ1 in an in vitro neutrophil chemotaxis assay (Fig. 5*C*), with 1 ng/mL TGFβ1 having an equivalent chemotactic effect as fMLP, a recognized neutrophil chemoattractant (Fig. 5*C*). Neutrophil chemotaxis toward 1 ng/mL TGFβ1 was significantly reduced when TGFβ-receptor signaling was blocked by preincubating neutrophils with ALK5 inhibitors, SB-525334 or Galunisertib compared with DMSO vehicle-treated controls (Fig. 5*D*).

We next verified whether platelet-derived TGFβ1 could induce human neutrophil migration. Significantly more neutrophils migrated toward thrombin-activated PRP than toward unstimulated PRP (Fig. 5*E*). This effect was reduced when neutrophils were pretreated with SB-525334 or Galunisertib ALK5 inhibitors compared with DMSO-treated controls (Fig. 5*E*). This shows that human neutrophil chemotaxis toward thrombin-activated PRP is partly attributable to the presence of platelet-derived TGFβ1. This reduced migratory response was mirrored using TGFβ1^fl/fl^.PF4-Cre PRP with murine bone-marrow-derived neutrophils (Fig. 5*F*). These novel findings suggest that platelet-derived TGFβ1 partially contributes toward neutrophil migration in vitro.

### Platelet-Derived TGFβ1 Does Not Significantly Induce Neutrophil Recruitment during Pulmonary Inflammation

**Figure 6.**
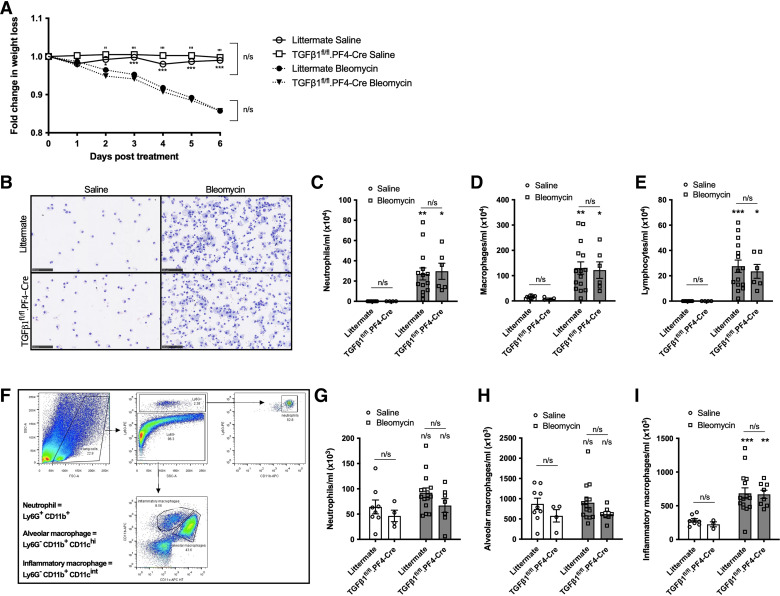
Platelet-derived TGFβ1 does not contribute to neutrophil recruitment during bleomycin-induced inflammation. *A*: TGFβ1^fl/fl^.PF4-Cre or littermates were given 50 IU bleomycin or saline (*n* = 9 littermate saline, *n* = 4 TGFβ1^fl/fl^.PF4-Cre saline, *n* = 15 littermate bleomycin, *n* = 8 TGFβ1^fl/fl^.PF4-Cre bleomycin) via an oropharyngeal route. Fold-change in body weight loss was monitored for 6 days post instillation. *B*: representative BALF cytospins after 6 days of treatment. Size denoted by 100-µm scale bar. Neutrophil (*C*), macrophages (*D*), or lymphocyte (*E*) populations from recovered BALF were quantified from cytospins (*n* = 9 littermate saline, *n* = 4 TGFβ1^fl/fl^.PF4-Cre saline, *n* = 15 littermate bleomycin, *n* = 8 TGFβ1^fl/fl^.PF4-Cre bleomycin). *F*: representative flow cytometric gating strategy to distinguish recovered cell populations from lung homogenate. Neutrophil (*G*), alveolar (*H*), or inflammatory macrophage cell (*I*) populations in lung homogenate were quantified by flow cytometry (*n* = 9 littermate saline, *n* = 4 TGFβ1^fl/fl^.PF4-Cre saline, *n* = 15 littermate bleomycin, *n* = 8 TGFβ1^fl/fl^.PF4-Cre bleomycin). Statistical differences were determined using one-way or two-way ANOVA with Holm–Sidak post hoc testing. In A, asterisks represent significance between treatments for littermate mice and apostrophes represent significance between treatments for TGFβ1^fl/fl^.PF4-Cre mice. In C and *E*, asterisks above the bars represent significance between saline or bleomycin group. Any other significant differences are indicated (n/s = not significant, *P < 0.05, **P < 0.01, ***P < 0.001). BALF, bronchoalveolar lavage fluid; IPF, idiopathic pulmonary fibrosis.

To address whether platelet-derived TGFβ1 can influence cellular recruitment during pulmonary inflammation in vivo, littermate or TGFβ1^fl/fl^.PF4-Cre mice were treated with 50 IU bleomycin. Untreated littermate and TGFβ1^fl/fl^.PF4-Cre mice have similar percentages of neutrophils in the lung or spleen (Supplemental Fig. S3), indicating that platelet-derived TGFβ1 does not affect neutrophil counts at baseline in naïve mice. During the peak of inflammation at *day 6*, littermate and TGFβ1^fl/fl^.PF4-Cre mice treated with bleomycin lost significantly more weight than saline-treated mice (Fig. 6*A*). Significantly increased numbers of neutrophils, macrophages, and lymphocytes were found in the BALF after bleomycin treatment compared with saline controls (Fig. 6, *B*–*E*). However, there was no significant difference between bleomycin-treated littermate or TGFβ1^fl/fl^.PF4-Cre mice (Fig. 6, *C*–*E*). Flow cytometry was used to quantify neutrophil, alveolar and inflammatory macrophage recruitment into the lung (Fig. 6, *F*–*I*). Bleomycin treatment led to significantly elevated inflammatory macrophages recruitment compared with saline controls (Fig. 6*I*), with equivalent numbers between littermate and TGFβ1^fl/fl^.PF4-Cre mice (Fig. 6*I*). To verify these results, we used another murine lung inflammation model based on inhaled LPS. No significant differences in cellular recruitment into the lung tissue or BALF were found between littermate and TGFβ1^fl/fl^.PF4-Cre mice after 6 h of LPS treatment (Supplemental Fig. S4). This suggests that although platelet-derived TGFβ1 influences neutrophil migration in vitro, it does not significantly drive cellular recruitment into the lung or airways in vivo.

### Platelet-Derived Mediators in IPF BALF May Contribute to Neutrophil Recruitment and Disease Severity

**Figure 7.**
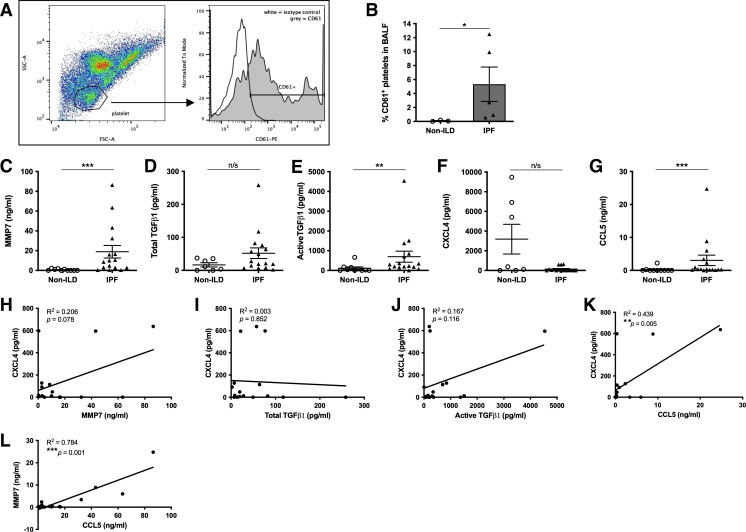
Platelet-derived mediators may contribute to neutrophil recruitment in IPF lung and disease severity. *A*: representative flow cytometric plot showing CD61 expression in platelet gate in IPF BALF. *B*: percentage of CD61^+^ platelets of total cells in BALF was quantified by flow cytometric analysis (*n* = 3 non-ILD and *n* = 5 IPF). MMP7 (*C*), total (*D*) or active TGFβ1 (*E*), CXCL4 (*F*) or CCL5 (*G*) concentrations were measured in BALF of patients with non-ILD and IPF (*n* = 7 and 16, respectively) by ELISA or MLEC bioassay. Concentrations of MMP7 (*H*), total (*I*) or active TGFβ1 (*J*), or CCL5 (*K*) in BALF of patients with IPF were correlated with paired CXCL4 (*n* = 16). *L*: concentrations of CCL5 in BALF of patients with IPF were correlated with paired MMP7 (*n* = 16). Statistical differences were determined using Mann–Whitney *U* tests or linear regression and any significant differences are indicated (n/s = not significant, *P < 0.05, **P < 0.01, ***P < 0.001). BALF, bronchoalveolar lavage fluid; IPF, idiopathic pulmonary fibrosis; MLEC, Mink lung epithelial cell bioassay; non-ILD, non-interstitial lung diseases.

As we previously observed an activated platelet signature of increasing CXCL4 concentrations significantly correlating with active TGFβ1 in IPF blood (Fig. 2), we next examined the BALF cellular composition in a cohort of patients with IPF and non-ILD (Supplemental Table S2). BALF from patients with IPF contained significantly more CD61^+^ platelets in BALF (Fig. 7, *A* and *B*) and elevated MMP7 and active TGFβ1, but not total TGFβ1 or CXCL4 concentrations compared with non-ILD controls (Fig. 7, *C*–*F*). The concentration of CCL5, a recognized neutrophil chemokine (37) secreted by activated platelets, was significantly increased in BALF from patients with IPF compared with non-ILD controls (Fig. 7*G*). MMP7 concentrations in IPF BALF also significantly correlated with increasing paired CXCL4 concentrations (Fig. 7*H*), suggesting that activated platelets in the BALF are linked with worse IPF disease severity. Total and active TGFβ1 did not correlate with CXCL4 (Fig. 7, *I* and *J*), suggesting that activated platelets may not represent the key source of this profibrotic and neutrophil chemotactic growth factor in the airways. However, we observed a significant positive correlation between CCL5 with either CXCL4 or MMP7 concentrations (Fig. 7, *K* and *L*). These data suggest that activated platelets may release CCL5 to mediate the observed neutrophilic infiltration into the BALF of patients with IPF (Fig. 5*B*).

In summary, we found platelet-derived TGFβ1 does not significantly drive pulmonary inflammation and fibrosis in the animal model of bleomycin-induced PF. Our patient studies have revealed that blood platelet counts do not consistently predict survival in IPF, even though an activated platelet signature correlating with active TGFβ1 was found in the blood of patients with IPF. Furthermore, activated platelets may not represent the predominant source of TGFβ1 in the airways but may drive human PF disease progression and neutrophil recruitment into the lungs through the release of mediators such as CCL5.

## DISCUSSION

Increased platelet reactivity and trapping within the lung have been previously described in IPF clinical and animal studies (22–24). Although these reports were unable to establish a direct causative relationship between platelets or platelet-derived products with IPF disease progression, the aim of this study was to determine the contribution of platelets and specifically, platelet-derived TGFβ1 in mediating lung inflammation and fibrosis in vivo.

Our finding of elevated MMP7 in the IPF plasma compared with non-ILD controls agrees with literature proposing MMP7 as an IPF plasma biomarker (36). Our initial clinical analysis showed a novel significant correlation between mortality and elevated platelet counts in our discovery cohort of patients with IPF. However, this observation was not reproduced in a separate larger validation cohort to confirm platelet counts as a prognostic biomarker in IPF. Based on data from the UK Biobank, we found that patients with IPF have significantly increased blood platelet counts compared with healthy controls—a finding that contrasts with results reported by Ntolios et al. (21). However, platelet to lymphocyte ratio (PLR) indices have been shown to be significantly increased in patients with IPF compared with controls (38) and patients with IPF with the greatest change in PLR have a poorer outcome (39). Therefore, analyzing blood platelet counts alone does not predict disease progression in patients with IPF and other platelet-related signatures such as PLR or PMV (21) should be considered.

Furthermore, our cohort of patients with IPF displayed an activated blood platelet signature consisting of CXCL4 concentrations correlating with increased active TGFβ1 concentrations, suggesting platelets may represent a cellular source of this profibrotic cytokine in the periphery of patients with IPF. Interestingly, we observed increased frequency of platelets and CXCR4 concentrations pairing with elevated MMP7 levels in the BALF from patients with IPF to suggest that activated platelets within the lung may play a role in IPF disease progression. However, concentrations of CXCR4 did not correlate with active TGFβ1 in IPF BALF. Further experiments examining activation markers, such as P-Selectin (CD62P) (21), on IPF BALF or blood platelets would be required to confirm these findings.

Recent evidence has proposed the lung as a major site for platelet biogenesis in mice with a distinct megakaryocyte subset, residing within the lung interstitium (40). In addition, lung megakaryocytes have also been proposed to have immune modulatory properties (41). Animal studies have indicated a pathogenic role for megakaryocytes in the bleomycin-induced model of PF by promoting fibroblast proliferation and differentiation (42). It is unknown whether megakaryopoiesis or the presence of interstitial megakaryocytes also occurs in human lungs, although megakaryocytes have been found in human lung capillaries (43). We readily detected platelets in the vasculature of lung and BALF from patients with IPF; however, we did not find any megakaryocytes within the blood vessels or interstitium of control or IPF lung.

PRP enhances fibroblast cell growth and contraction of collagen matrix (44, 45), although the relative contribution of platelet-derived TGFβ1 has not been investigated fully. Platelets were readily detected in bleomycin-treated littermate and TGFβ1^fl/fl^.PF4-Cre lungs; however, the degree of fibrosis was equivalent in both treatment groups, suggesting that platelet-derived TGFβ1 is redundant in this animal model of PF. Of importance, it should be noted that although bleomycin remains the gold standard animal model for PF research, it does not fully recapitulate the human disease. The degree of lung fibrosis in mice is dependent on bleomycin dose (46) and collagen degrades over time after bleomycin treatment (47), whereas IPF is a nonresolving progressive disease. Therefore, the contribution of platelet-derived TGFβ1 in mediating human IPF disease remains unknown, although global depletion of either platelets or TGFβ1 is not feasible therapeutic options for patients with IPF.

Activated platelets secrete over 300 proteins (48) and PRP contains key profibrotic growth factors such as platelet-derived growth factor (PDGF) (49). Therefore, it is conceivable that other growth factors such as PDGF released by activated platelets may play a dominant profibrotic role in the absence of platelet-derived TGFβ1 in TGFβ1^fl/fl^.PF4-Cre mice. Studies focused on the profibrotic role of PGDF in IPF have shown that inhibiting PDGF-BB signaling with Imatinib (a tyrosine kinase inhibitor of PDGFR) or APB5 (a PDGFR-β blocking antibody) attenuates bleomycin-induced PF (50, 51). Furthermore, Nintedanib, an approved antifibrotic treatment for IPF, is a small molecule inhibitor against receptor tyrosine kinases such as PDGFR (52), which inhibits fibroblast proliferation and differentiation and ECM production in vitro. Therefore, the study of other platelet-derived mediators in driving PF pathology still remains an important research question.

We have shown PRP is a potent neutrophil chemoattractant and platelet-derived TGFβ1 partially contributed to neutrophil migration in vitro. TGFβ1 has previously been reported as a neutrophil chemoattractant (53), but to our best knowledge, this is the first instance that platelet-derived TGFβ1 has been linked to neutrophil chemotaxis. We observed no difference in the number or percentage of neutrophils recruited into the lung or BALF of littermate or TGFβ1^fl/fl^.PF4-Cre mice after bleomycin or LPS challenge. This suggests that platelet-derived TGFβ1 has a redundant role in neutrophil migration in these in vivo models. CXCL5 and CXCL7 are important platelet-derived neutrophil chemoattractants in a murine metastasis model (54). It is probable that these or other platelet-derived neutrophil chemoattractants, such as CCL5 (55), CXCL4 (56), or serotonin (57), could compensate for the absence of TGFβ1 in TGFβ1^fl/fl^.PF4-Cre mice in neutrophil recruitment. Furthermore, we found significantly increased CCL5 concentrations in the BALF from patients with IPF, which positively correlated with CXCL4 and MMP7 levels. This suggests that activated platelets could be secreting other chemokines such as CCL5 in the lung, which may be important to drive neutrophil infiltration to mediate lung damage in IPF.

Overall, the novel findings we present here offer a better understanding of platelets and platelet-derived TGFβ1 in both human IPF disease and animal models of PF. Further studies investigating the cellular effects of other platelet-derived mediators, such as chemokines and profibrotic growth factors, will help to facilitate a focused cell-targeted approach to developing novel treatments for patients with IPF.

## DATA AVAILABILITY

Data will be made available upon reasonable request.

## SUPPLEMENTAL DATA

10.6084/m9.figshare.22259326.v1 Supplemental Methods, Supplemental Figs. S1–S4, and Supplemental Tables S1 and S2: https://doi.org/10.6084/m9.figshare.22259326.v1.

## GRANTS

This work was undertaken at UCLH/UCL, which received a proportion of the funding from the Department of Health’s NIHR Biomedical Research Centres funding scheme. This work was also supported by the Medical Research Council (MRC; grant codes G0800340 and MR/K004158/1), a European Commission FP7 award PIEF GA-2012–326928 to C. Rebeyrol, and a GW4 BioMed MRC Doctoral Training Partnership award to A. Duckworth.

## DISCLOSURES

No conflicts of interest, financial or otherwise, are declared by the authors.

## AUTHOR CONTRIBUTIONS

D.L.W.C., C.J.S., and J.C.P. conceived and designed research; D.L.W.C., T.A.M., J.S., C.R., H.S.G., H.L.B., M.H., E.K.D., R.J.J., A.A.K., A.D., M.L., and C.J.S. performed experiments; D.L.W.C., T.A.M., J.S., E.K.D., and A.D. analyzed data; D.L.W.C., T.A.M., J.S., E.K.D., C.J.S., and J.C.P. interpreted results of experiments; D.L.W.C. and T.A.M. prepared figures; D.L.W.C. and T.A.M. drafted manuscript; D.L.W.C., T.A.M., J.S., C.R., H.S.G., H.L.B., M.H., E.K.D., R.J.J., A.A.K., A.D., M.L., C.J.S., and J.C.P. edited and revised manuscript; D.L.W.C., T.A.M., J.S., C.R., H.S.G., H.L.B., M.H., E.K.D., R.J.J., A.A.K., A.D., M.L., C.J.S., and J.C.P. approved final version of manuscript.
